# Effect of Monotherapy with Darunavir/Ritonavir on Viral Load in Seminal Fluid, and Quality Parameters of Semen in HIV-1-Positive Patients

**DOI:** 10.1371/journal.pone.0159305

**Published:** 2016-07-21

**Authors:** Miguel A. Lopez-Ruz, Purificación Navas, Miguel A. López-Zúñiga, María Carmen Gonzalvo, Antonio Sampedro, Juan Pasquau, Carmen Hidalgo-Tenorio, Rosario Javier, José A. Castilla

**Affiliations:** 1 Unidad de Enfermedades Infecciosas, Hospital Virgen de las Nieves, Complejo Hospitalario Universitario Granada, Instituto de Investigación Biosanitaria de Granada (IIBG), Granada, Spain; 2 Unidad Reproducción, UGC Laboratorio Clínico y UGC Obstetricia y Ginecología, Complejo Hospitalario Universitario Granada, Instituto de Investigación Biosanitaria de Granada (IIBG), Granada, Spain; 3 Dpto. Anatomía y Embriología Humana, Programa del Doctorado de Biomedicina Regenerativa, Universidad de Granada, Granada, Spain; 4 Unidad de Microbiología, Hospital Virgen de las Nieves, Complejo Hospitalario Universitario Granada, Instituto de Investigación Biosanitaria de Granada (IIBG), Granada, Spain; UCL, UNITED KINGDOM

## Abstract

Patients with human immunodeficiency virus type 1 (HIV-1) who receive antiretroviral therapy (ART) often achieve increased survival and improved quality of life. In this respect, monotherapy with darunavir/ritonavir (mDRV/r) can be a useful treatment strategy. This prospective study analyses the effect of mDRV/r on sperm quality and viral load in a group of 28 patients who had previously been given conventional ART and who had recorded a viral load <20 copies/mL for at least six months. These patients were given mDRV/r at a dose of 800/100 mg for 48 weeks. At baseline (V0), CD4, CD8, FSH, LH and testosterone levels were measured, together with HIV-1 viral load in plasma and semen. In addition, seminal fluid quality was studied before mDRV/r treatment was prescribed. At week 48 (V1), HIV-1 viral load in plasma and semen and the quality of the seminal fluid were again measured. The results obtained indicate that at V0, 10% of the patients with ART had a positive viral load in seminal fluid (>20 copies/ml), and that at V1, after mDRV/r treatment, this figure had fallen to 3%. The quality of seminal fluid was close to normal in 57% of patients at V0 and in 62% at V1. We conclude that, similar to ART, mDRV/r maintains HIV-1 viral load in most patients, and that there is no worsening in seminal fluid quality.

## Introduction

The principal route of transmission of the human immunodeficiency virus (HIV-1) is heterosexual activity. However, the risk of transmission from HIV-1 males who have successfully completed a course of antiretroviral therapy (ART) is close to zero (1:100000) if the plasma viral load has been undetectable during the previous six months, if the patient has adhered strictly to the ART and if no other sexually transmitted disease is present [1].

Sexual transmission between partners is significantly reduced when the seropositive member receives highly active antiretroviral therapy (HAART) [2,3]. Some authors have observed decreased seminal fluid parameters in patients with HIV-1 [4–7], but others have found no significant differences in this respect [8]. In patients who began treatment with HAART, a reduction in the percentage of motile sperm after 48 weeks of follow-up was observed [9]. Decreased mitochondrial DNA in the sperm of patients treated with nucleoside reverse transcriptase inhibitors has been reported [10,11].

Treatment guidelines for patients with HIV-1 recommend ART [12,13]. However, due to possible side effects from the nucleotides or nucleosides that form part of this therapy, it has been suggested that these should be replaced by protease inhibitors in monotherapy, such as lopinavir/ritonavir (LPV/r) [14,15] or DRV/r [16]. Both drugs can be used safely in patients with suppressed viral load, without resistance against LPV [15] or DRV [17], and with an efficacy slightly lower than that achieved by ART [18]. No immune-activation has been observed during episodes of transient viremia (blips) in patients with mDRV/r [19] nor has any replication of the HIV-1, such as proviral-DNA, been seen during blips with mDRV/r [20]. According to extended follow-up observation, good results are achieved and maintained with mDRV [21] or with LPV/r in monotherapy [22].

It has been shown that monotherapy with LPV [23] or with DRV [24] can reverse certain secondary effects, attributable to nucleosides, resulting from mitochondrial DNA damage. Accordingly, for some cases treatment with protease inhibitors in monotherapy has been recommended [12,13]. No differences at the neurocognitive level have been observed between ART and monotherapy with protease inhibitors [25].

Studies have analysed monotherapy with LPV/r [26] and DRV/r [27], but none have investigated semen quality in this respect. DRV in seminal fluid was measured in a study with 10 patients receiving ART with DRV/r (800/100 mg), following previous therapy with antiretrovirals and other protease inhibitors; in this group, the DRV concentration was above EC50 for wild-type in nine of the ten patients [28]. Moreover, some had above-EC50 levels for resistant virus [29]. In another study, the levels of darunavir observed in seminal fluid were 200 times greater than the EC90 of the HIV-1 [30].

The aim of the present study was to evaluate the changes produced in the quality and viral load of seminal fluid in HIV-1 patients who had previously received ART, and who were then treated with mDRV/r for 48 weeks. Concurrently, we evaluated the viral load in seminal fluid and other parameters of semen quality in patients receiving ART, seeking to relate these parameters to CD4 lymphocytes and the CD4 nadir.

## Patients and Method

HIV-1 patients were drawn from those attending the outpatient infections clinic at Virgen de las Nieves university hospital (HUVN) (Granada, Spain). These patients were receiving stable conventional ART, including two analogues of nucleotides and a third drug. All had undetectable viral load (<20 copies/mL). All these patients provided signed informed consent to participate in the study. Those with active sexually transmitted diseases at the time of sample collection were excluded. The study was approved by the hospital’s ethics committee. The patients’ data were codified to maintain anonymity, in accordance with Spanish data protection laws.

### Inclusion criteria

- Males infected with HIV-1, with undetectable plasma viral load (<20 copies/mL) for at least six months;
- At least 18 years of age.

### Exclusion criteria

- Patients not adhering to scheduled outpatient appointments;
- Patients with a history of endocrinological or genital treatment or disease that could affect semen quality.

### Study design

Prospective, observational study over 48 weeks, with determination at baseline (V0) and at week 48 (V1) of follow-up. All patients were informed of the nature of the study prior to the first sample acquisition. The sample was obtained by masturbation following 3–4 days of sexual abstinence.

The outpatient visits were scheduled for each patient every four months. Adherence to the medication was evaluated by checking with the hospital’s pharmacy regarding the patient’s prescription record, and by questioning the patient in the outpatient clinic.

### Variables analysed

The parameters recorded were age, alcohol and tobacco consumption, androgenic history, reproductive history, CD4 count, CD4 nadir, type of ART, HIV-1 viral load in plasma and seminal fluid. The parameters considered in the semen quality analysis were viscosity, visual aspect, liquefaction, volume, pH, total sperm count, percentage of sperm with progressive motility and percentage of live sperm with normal forms. The teratozoospermia index was calculated. Serology was performed for hepatitis B and C, FSH, LH and testosterone.

### Semen analysis

Most of the samples were collected in the laboratory of the Reproduction Unit at HUVN. Less than 5% of the samples were collected by the participants in their own homes. In the latter case, the semen sample was maintained at body temperature and delivered to the laboratory within 45 minutes. On receipt of the sample in the laboratory, the following data were recorded: period of abstinence from sexual activity, whether any fraction of the ejaculate was lost, drugs taken, recent fever, body mass index and any recent participation in extreme sports. All samples were analysed within one hour of collection. Analyses were performed by a single, well-trained investigator with over 10 years’ experience and who regularly participates in such studies. This procedure minimised inter-individual variation in analyses and maximised the precision of the study.

Each participant provided four semen samples: two before starting monotherapy and another two after 48 weeks. The interval between the first and the second of each pair of samples was 15 days.

The ejaculates were analysed in accordance with WHO recommendations [31] and with international recommendations for the study of seminal fluid quality [32]. Sperm concentrations and those of round cells were determined using an improved Neubauer counting chamber and positive-displacement pipettes. The total number of sperm was calculated by multiplying the sperm count by the seminal fluid volume which, in turn, was measured as sample weight. Sperm motility was evaluated visually and expressed as a percentage. The percentage of those participants whose values were observed to be below the lower limit of reference values (WHO 2010) for sperm parameters was calculated.

### Determination of viral load in seminal liquid

Two fractions were generated by centrifugation (3000 g, 15 min). Real time polymerase chain reaction (RT-PCR) was used to quantify RNA HIV-1 in the final sample, applying a technique adapted for HIV-1 in the COBAS analyser (Ampliprep/Taqman assay version 2.0; Roche).

Two determinations were performed on seminal fluid samples acquired 15 days apart, before commencing monotherapy. A further two were obtained following the conclusion of the monotherapy administration, separated by the same interval (four samples in total per patient).

### Statistical analyses

Descriptive analyses were performed on the principal variables collected. For quantitative variables, measures of central tendency (mean and median) and of dispersion (standard deviation and range) were calculated. For the qualitative variables, absolute and relative frequencies were calculated. Differences in pre and post- monotherapy values, in relation to semen quality, were evaluated using the McNemar test for the qualitative variables and the Student *t*-test for related quantitative variables. The Wilcoxon test was used when the distribution was non-normal. The concentrations and total numbers of sperm, which presented a non-normal distribution (non-Gaussian), were log transformed to approximate a normal distribution [32].

The level of statistical significance was set at p<0.05. IBM SPSS 19 statistical software was used throughout.

## Results

### Participants

**Fig 1:**
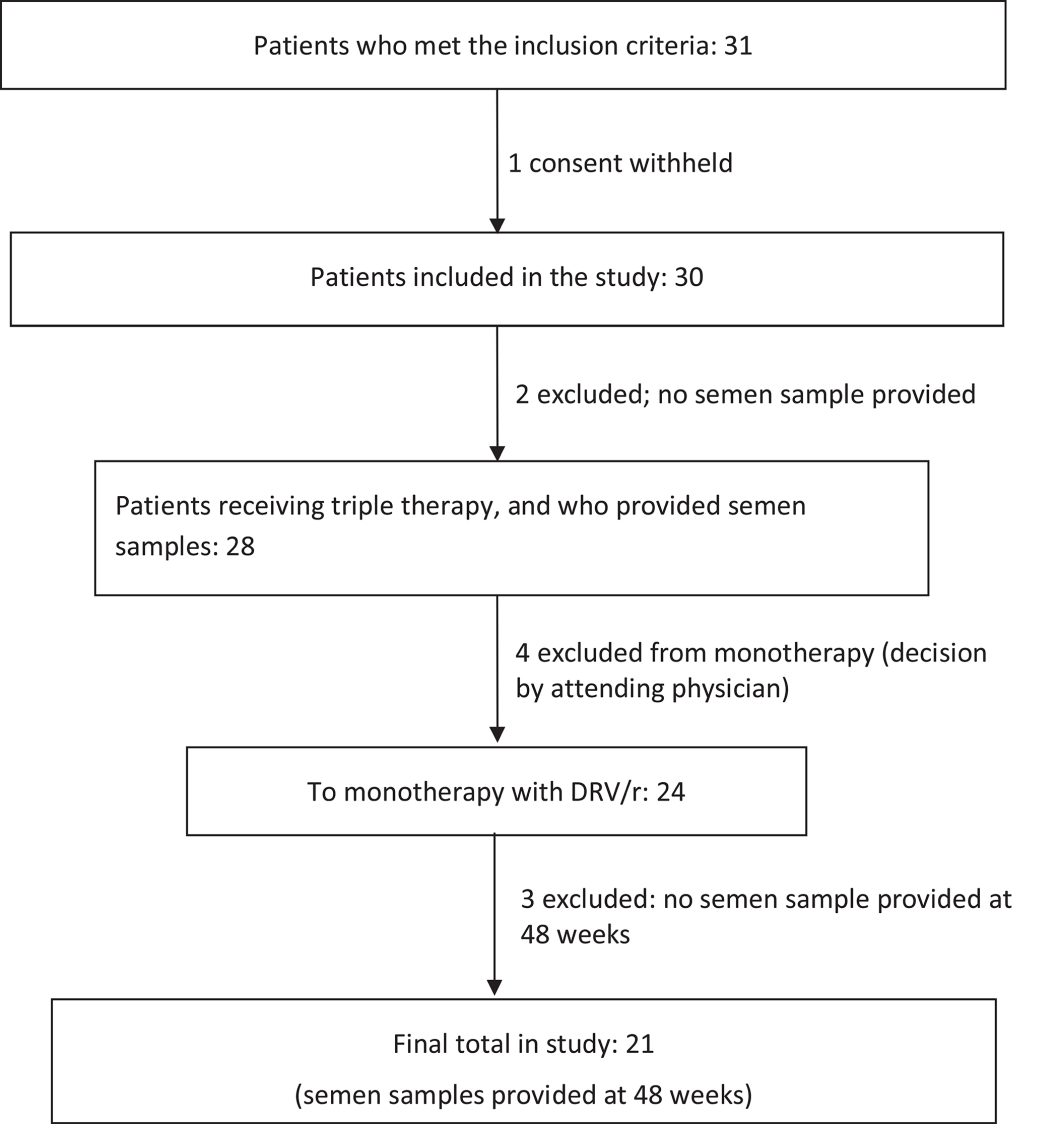
Flow of patients through the study.

Of the 31 patients who met the selection criteria and were initially selected, 28 were recruited to the study. Subsequently, three were excluded (one who refused consent and two who failed to donate semen). Of the remaining 28 patients, four did not enter the monotherapy phase (on the advice of the attending physician), although they did provide semen samples at the baseline visit. Three failed to provide a semen sample at 48 weeks, and thus 21 patients with mDRV/r finally completed the 48-week follow-up. No patient left the study due to positive viral load in plasma, or because of side effects (Fig 1).

### General characteristics of the patients included in the study (V0)

At baseline, 28 patients were included in the study. The mean age was 41 years (SD: 9.3), the median CD4 value was 659/mL (range: 230–1242) and the median CD4 nadir was 270/mL (range: 10–634).The ART treatment is summarised in Table 1.

**Table 1:** ART scheme at V0.

Original treatment type	Initial sample; Initial treatment^a^	Final sample; Initial treatment
TDF+FTC+efavirenz	12	10
TDF+FTC+etravirine	1	-
TDF+FTC+raltegravir	2	2
TDF+FTC+DRN/r	3	2
TDF+FTC+atazanavir	1	1
TDF+FTC+nevirapine	1	-
TDF+FTC+atazanavir/r	2	2
ABC+3TC+atazanavir/r	1	1
ABC+3TC+DRV/r	3	2
ABC+3TC+Lopinavir/r	2	1
Total	28	21

Notes

^a^ Numbers represent the numbers of patients for each kind of initial treatment before the monotherapy.

TDF: tenofovir; FTC: emtricitabine; DRN/r: darunavir/ritonavir; ABC: abacavir; 3TC: lamivudine.

### Baseline results (V0)

#### Viral load in seminal fluid

At V0, HIV-PCR in the seminal fluid was analysed on the first sample provided by 27 patients (one was not processed because of PCR inhibition); four (15%) were positive (>20 copies/mL), with a range of 35–1210 copies/mL.

The second HIV-PCR analysis in seminal fluid at V0 was performed on 22 samples from 28 patients (six were unsuccessful because of PCR inhibition); one (5%) was positive, with 399 copies/mL.

At V0, 5 of the 49 samples (10%) presented HIV-1-positive viral load in seminal fluid.

#### Semen quality in the patients on ART (V0)

The data on semen quality in patients on ART (V0) are summarised in Table 2. Only 16 of the 28 samples (57.1%) patients at V0 presented higher sperm values than the lower limit of the WHO 2010 reference values. The alterations most frequently observed were oligozoospermia and teratozoospermia. No significant differences were observed in the sperm parameters analysed at V0 and V1 (Table 3).

**Table 2:** Semen quality in patients receiving ART and mDRV/r.

**Semen quality: ART**	**V0: n = 28**	**V1: n = 21**
Normal	16 (57.1%)	13(61.9%); p = 0.755
Oligoteratospermia	2 (7.1%)	2(9.6%)
Oligoastenospermia	2 (7.1%)	1(4.8%)
Oligoteratoastenospermia	3 (10.7%)	1(4.8%)
Teratozoospermia	4 (14.3%)	1(4.8%)
Astenozoospermia	0 (0%)	2(9.6%)
Oligozoospermia	1 (3.6%)	1(4.8%)

**Table 3:** Seminal fluid values of the 21 patients who completed the study.

	V0 ^a^Median (Q1-Q3)	V1Median (Q1-Q3)	p
Volume; mL	2.3(1.7–3)	1.9(1.5–3.37)	0.146
pH	7.5(7.2–7.5)	7.5(7.2–7.7)	0.976
Sperm; x10^6^/mL	59(20.5–141.5)	39(14–109.5)	0.068
Round cells; x10^6^/mL	3(1–8)	2(1.12–3.75)	0.178
Total sperm count; x10^6^	154(42–319.5)	132(31.8–259.5)	0.117
Motility; %	45(32.5–62.5)	52.5(40–65)	0.755
Motility; total	60(42–70)	60(45–709)	0.657
Normal structure	5(3–6)	4(4–7.5)	0.388
Live sperm; %	88(79.5–90.5)	86.5(80.5–90.75)	0.176
Teratospermia index	1.3(1.2–1.4)	1.3(1.2–1.4)	0.950

^a^ Mean values of the two samples analysed at V0 and V1.

### Results at 48 weeks (V1)

#### Serological and hormonal status

No change was observed in serostatus against hepatitis B or C, or in the hormonal profile studied (FSH, LH and testosterone).

#### Viral load in seminal fluid at week 48

In the first seminal fluid sample, only 1 of 17 patients (6%) had a positive viral load (139 copies/mL). No results were obtained from the remaining four patients, due to PCR inhibition.

With respect to the second sample, none of the 15 patients analysed had a positive viral load (of the total of 21 patients; no results were obtained from six, due to PCR inhibition). Of the total sample of 32 mDRV/r patients, only one (3%) was positive. This patient was also positive at V0. The percentage of semen samples with a positive viral load was similar in monotherapy and in tri-therapy (3% vs. 10%) and no statistically significant differences were observed (p = 0.3946).

#### Semen quality at week 48 (V1)

Table 2 summarises the results obtained at V1 (48 weeks). No statistically significant differences were observed in the percentage of patients with sperm values above than the lower limit of the WHO 2010 reference values. As shown in Table 3, there were no statistically significant differences between the seminal parameters at V0 and V1 in the patients who completed the study.

### Bivariate analyses

With respect to semen sample quality, no statistically significant associations were observed at V0. This applied not only to CD4 levels (p = 0.781) but also to the CD4 nadir (p = 0.3). At V1, too, there were no statistically significant associations in the semen quality with respect to CD4 levels (p = 0.750) or the CD4 nadir (p = 0.595).

## Discussion

The aim of this study was to evaluate the viral load in the genital compartment, a possible sanctuary site, following treatment with mDRV/r, and to determine whether this treatment, extended to include nucleos(t)ide analogues, improved seminal fluid quality in HIV-1-positive patients.

The mDRV/r treatment combination was adopted because of its potency and ease of administration and because our team, as well as others, had considerable experience in its use [16].

The seminal fluid viral load of the patients receiving ART was analysed in 49 samples (in the remaining cases, no such analysis was possible, due to PCR inhibition). These samples were obtained from 27 of the 28 patients who began the study; for one patient, it was not possible to determine the viral load in any of the semen samples provided, due to PCR inhibition. Of these 49 samples, only five (10%) were positive (>20 copies/mL), with the viral load ranging from 35 to 1210 copies/mL. These values are slightly lower than those observed by Torres-Cornejo *et al*. [33]. Of the 28 initial patients, 21 completed the 48-week follow-up and provided two samples each. Viral load was determined in 32 of 42 samples provided. The 3% positivity obtained with mDRV/r is lower than the 16% positivity in monotherapy with protease inhibitors observed by Torres-Cornejo *et al*. [33] and the 20% reported by Gutmann *et al*. [34], using LPV/r in monotherapy. Other studies have obtained results similar to ours; thus, Ghosn *et al*. [26] reported 0%, using LPV/r in monotherapy, and Lambert-Niclot *et al*. [27] obtained 4%, using mDRV/r at a dose of 600/100 b.i.d. The latter two studies employed viral load detection limits of 100 and 200 copies/mL, respectively, which were higher than the system used in the present study.

Only one patient receiving mDRV/r treatment had a positive viral load of 139 copies/mL, which cannot be compared with the viral load values of the patients receiving ART. This patient presented a positive viral load in seminal fluid while receiving ART.

These findings of a very low percentage of positive viral load with mDRV/r in seminal fluid lead us to believe that this type of therapy is as safe as ART, with respect to levels of viral load seminal fluid. In the central nervous system, similarly good results have been reported for mDRV/r [25, 35, 36]. Monotherapy with protease inhibitors has been shown to produce results to comparable with those of ART in other body compartments, such as lymphatic tissue [37].

With respect to the quality of seminal fluid, after discarding endocrinological and genital abnormalities (data not shown), only 57% of the patients receiving ART surpassed the lower limit of the WHO 2010 reference values for semen quality [31] (see Table 3). These data confirm the poor quality of semen of HIV-1 patients–with or without treatment [5, 6]–and the negative influence of this condition on the quality of seminal fluid in patients commencing ART [9]. The reasons for this poor seminal fluid quality are unclear, but presumably it results from the HIV-1 infection itself [38], or the damage attributable to the retroviral therapy [39], specifically, the mitochondrial damage caused by nucleoside analogues [10].

Of the patients who proceeded to receive mDRV/r, the percentage with normal semen rose to 62%, although the difference, as regards the overall quality of the semen, was not statistically significant (Table 3). The maintenance of sperm quality observed in the study could be due to the analogues being received by the ART patients (Table 1), as these analogues are less associated with mitochondrial toxicity [39, 40, 41]. Although a decrease in sperm concentration was observed between V0 and V1, when the total number of ejaculated sperm were taken into account, this decrease was less marked. According to the WHO semen analysis manual, this latter parameter is of most clinical value in the study of spermatogenesis.

According to our literature review, no previous studies have been conducted to compare changes in seminal fluid quality after patients proceed from ART to mDRV/r treatment, or to LPV/r in monotherapy.

In our study, neither in the ART patients nor in those receiving mDRV/r were any statistically significant relationships observed between CD4 levels and seminal quality. Neither were there significant relationships between the CD4 nadir and semen quality.

In view of the results obtained in the present study, we conclude that the viral load in seminal fluid has the same characteristics in ART patients as in those receiving mDRV/r, and that there was no worsening of semen quality relative to baseline. The possibility of changes in the cells and molecules of the immune system, with respect to sperm, cannot be discounted and this question needs to be analysed in future studies. The results we report could provide clinicians with guidance in providing an appropriate response to the reproductive desires of the seronegative partner of an HIV-1-positive patient [42]. Nevertheless, the present findings need to be confirmed in studies with larger numbers of patients.

## References

[1] Vernazza P, Hirshel B, Bernasconi E, Flepp M. Les personnes seropositives ne souffrant d’aucune autre MST et suivant un traitement antiretroviral efficace ne transmettent pas le HIV par voie sexuelle. Bulletin des Médecins Suisses 2008; 89:165–169.

[2] Del Romero J, Castilla J, Hernando V, Rodríguez C, García S. Combined antiretroviral treatment and heterosexual transmission of HIV-1: cross sectional and prospective cohort study. BMJ. 2010;340:c2205 10.1136/bmj.c2205 20472675 PMC2871073

[3] Barreiro P, del Romero J, Leal M, Hernández V, Asensio R, De Mendoza C, et al Natural pregnancies in HIV-serodiscordant couples receiving successful antiretroviral therapy. J Acquir Immune Defic Syndr. 2006;43:324–326. 17003695 10.1097/01.qai.0000243091.40490.fd

[4] Bujan L, Sergerie M, Moinard N, Martinet S, Porte L, Massip P, et al Decreased semen volume and spermatozoa motility in HIV-1-infected patients under antiretroviral treatment. J Androl. 2007;28:444–452. 17215546 10.2164/jandrol.106.001529

[5] Pilatz A, Discher T, Lochnit G, Wolf J, Hans-Christian S, Schuttler C, et al Semen quality in HIV patients under stable antiretroviral therapy is impaired compared to WHO 2010 reference values and on sperm proteome level. AIDS 2014;28:875–880. 24614089 10.1097/QAD.0000000000000161

[6] Kehl S, Weigel M, Muller D, Gentili M, Hoermann A, Sutterlin M. HIV-infection and moderm antiretroviral therapy impair sperm quality. Arch Gynecol Obstet. 2011;284: 229–233. 10.1007/s00404-011-1898-6 21448708

[7] Lorusso F, Palmisano M, Chironna M, Vacca M, Masciandaro P, Bassi E, et al Impact of chronic viral diseases on semen parameters. Andrologia 2010;42:121–126. 10.1111/j.1439-0272.2009.00970.x 20384803

[8] Van Leeuven E, Wit FW, Prins JM, Reiss P, van der Veen F, Repping S. Semen quality remains stable during 96 weeks of untreated human immunodeficiency virus-1 infection. Fertil Steril. 2008;90:536–541. 10.1016/j.fertnstert.2007.06.102 18023441

[9] Van Leeuven E, Wit F, Repping S, Eeftinck JK, Reiss P, van der Veen F, et al Effects of antiretroviral therapy on semen quality. AIDS 2008;22:637–642. 10.1097/QAD.0b013e3282f4de10 18317005

[10] Pavili L, Daudim M, Moinard N, Walschaerts M, Cuzin L, Massip P, et al Decrease of mitochondrial DNA level in sperm from patients infected with human immunodeficiency virus-1 linked to nucleoside analogue reverse transcriptase inhibitors. Fertil Steril. 2010;94:2151–2156. 10.1016/j.fertnstert.2009.12.080 20153854

[11] Dielhl S, Vernazza P, Trein A, Schnaitmann E, Grimbacher B, Bernhard S, et al Mitochondrial DNA and sperm quality in patients under antiretroviral therapy. AIDS 2003;17:450–451. 12556705 10.1097/00002030-200302140-00025

[12] Documento de Consenso de Gesida/Secretaria del Plan Nacional sobre el SIDA sobre el tratamiento antirretroviral del adulto (Updated January 2015). Available: http://gesida.seimc.org/pcientifica/dcconsensos.asp.

[13] Guías Clínicas. EACS. November 2014.Version 7.1. Available: http://onusida.org.co/documentos/eacsguidelinesSpanish5-4.pdf.

[14] Pulido F, Arribas JR, Delgado R, Cabrero E, González-García J, Pérez-Elias MJ, et al OK04 Study Group. Lopinavir-ritonavir monotherapy versus lopinavir-ritonavir and two nucleosides for maintenance therapy of HIV. AIDS 2008;22(2):F1–9. 18097218 10.1097/QAD.0b013e3282f4243b

[15] Nunes EP, Santini de Oliveira M, Merçon M, Zajdenverg R, Faulhaber JC, Pilotto JH, et al Monotherapy with lopinavir/ritonavir as maintenance after HIV-1 viral suppression: results of a 96-week randomized, controlled, open-label, pilot trial (KalMo study). HIV Clin Trials 2009;10:368–374. 10.1310/hct1006-368 20133267

[16] Arribas JR, Horban A, Gerstoft J, Fätkenheuer G, Nelson M, Clumeck N, et al The MONET trial: darunavir/ritonavir with or without nucleoside analogues, for patients with HIV RNA below 50 copies/ml. AIDS 2010;24:23–30. 10.1097/QAD.0b013e3283348944 20010070

[17] Pulido F, Arribas J, Hill A, Moecklinghoff C. No evidence for evolution of genotypic resistance after three years of treatment with darunavir/ritonavir, with or without nucleoside analogues. AIDS Res Hum Retroviruses 2012;28:1167–1169. 22380531 10.1089/AID.2011.0256

[18] Mathis S, Khanlari B, Pulido F, Schechter M, Negredo E, Nelson M, et al Effectiveness of protease inhibitor monotherapy versus combination antiretroviral maintenance therapy: a meta-analysis. PLoS One 011;6(7):e22003. 10.1371/journal.pone.0022003 PMC3139616 21811554

[19] Benmarzouk-Hidalgo O, Torres-Cornejo A, Gutiérrez A, Ruiz R, Viciana P, López Cortes L. Immune activation throughout a boosted darunavir monotherapy simplification strategy. Clin Microbiol Infect. 2014;20:1297–1303. 10.1111/1469-0691.12521 24372830

[20] Torres-Cornejo A, Benmarzouk-Hidalgo O, Gutiérrez A, Pérez P, Martin Peña R, Ruiz RP, et al Cellular HIV reservoir replenishment is not affected by blip or intermittent viremia episodes during darunavir/ritonavir monotherapy. AIDS 2014;28:201–208. 10.1097/QAD.0000000000000060 24361681

[21] Clumeck N, Rieger A, Banhegyi D, Schmidt W, Hill A, Van Delft Y, et al 96 week results from the MONET trial: a randomized comparison of darunavir/ritonavir with versus without nucleoside analogues, for patients with HIV RNA <50 copies/mL at baseline. J Antimicrob Chemother. 2011;66:1878–1885. 10.1093/jac/dkr199 21652619

[22] Arribas JR, Delgado R, Arranz A, Muñoz R, Portilla J, Pasquau J, et al OK04 Study Group. Lopinavir-ritonavir monotherapy versus lopinavir-ritonavir and 2 nucleosides for maintenance therapy of HIV: 96-week analysis. J Acquir Immune Defic Syndr. 2009;51:147–152. 10.1097/QAI.0b013e3181a56de5 19349870

[23] Cameron DW, da Silva BA, Arribas JR, Myers RA, Bellos NC, Gilmore N, et al A 96 week comparison of lopinavir-ritonavir combination therapy followed by lopinavir/ritonavir monotherapy versus efavirenz combination therapy. J Infec Dis. 2008;198:234–240. 18540803 10.1086/589622

[24] Valantin MA, Kolta S, Flandre P, Algarte M, Meynard JL, Ponscarme D, et al Body fat distribution in HIV infected patients treated for 96 weeks with darunavir/r monotherapy vs. darunavir/r plus nucleoside reverse transcriptase inhibitors: the MONOI-ANRS 136 sub-study. HIV Medicine 2012;13:505–515. 10.1111/j.1468-1293.2012.01004.x 22416798

[25] Pérez-Valero I, González A, Estebánez M, Monje S, Montes ML, Bayon C, et al A prospective cohort study of neurocognitive function in aviremic HIV infected patients treated with 1 or 3 anti-retrovirals. Clin Infect Dis. 2014;59:1627–1634. 10.1093/cid/ciu640 25114032 PMC4650773

[26] Ghosn J, Chaix ML, Peytavin G, Bresson JL, Galimand J, Girard PM, et al Absence shedding in male genital tract after 1 year of first line lopinavir/ritonavir alone or in combination with zidovudine/lamivudine. J Antimicrobial Chemotherapy 2008;61:1344–1347. 10.1093/jac/dkn098 18343806

[27] Lambert-Niclot S, Peytavin G, Duvivier C, Poirot C, Algarte-Genin M, Pakianather S, et al Low frequency of intermittent HIV-semen excretion in patients treated with darunavir-ritonavir 600/100 mg twice a day plus two nucleoside reverse transcriptase inhibitors or monotherapy. Antimicrobial Agents and Chemotherapy 2010;54:4910–4913. 10.1128/AAC.00725-10 20713677 PMC2976146

[28] Ghosn J, Slama L, Chermak A, Houssaini A, Lambert-Niclot S, Schneider L, et al Switching to darunavir/ritonavir 800/100mg once-daily containing regimen maintains virological control in fully suppressed pre-treated patients infected with HIV-1. J Medical Virology 2013;85:8–15. 10.1002/jmv.23404 23024008

[29] Taylor S, Jayasuriya A, Berry A, Gilleran G, Duffy N, Else L, et al Darunavir concentrations exceed the protein-corrected EC50 for wild-type HIV in the semen of HIV 1-infected men. AIDS 2010;24:2583–2586. 10.1097/QAD.0b013e32833ead18 20736813

[30] Antoniu T, Hasan S, Loutfy M, Kovacs C, Brunetta J, Smith G, et al Pharmacokinetics of maraviroc, raltegravir, darunavir, and etravirine in the semen of HIV-infected Men. J Acquir Immune Defic Syndrome 2013;62:e58–e60. 10.1097/QAI.0b013e31827a0d71 23328092

[31] Cooper TG, Noonan E, von Eckardstein S, Auger J, Baker HW, Behre HM, et al World Health Organization reference values for human semen characteristics. Human Reprod Update 2010;16:231–245. 10.1093/humupd/dmp048 19934213

[32] Sánchez-Pozo MC, Mendiola J, Serrano M, Mozas J, Björndahl L, Menkveld R, et al Proposal of guidelines for the appraisal of SEMen QUAlity studies (SEMQUA). Hum Reprod. 2013;28:10–21. 10.1093/humrep/des355 23054068

[33] Torres-Cornejo A, Benmarzouk Hidalgo O, Viciana P, Sánchez-Sanchez B, López-Ruz MA, López-Cortés LF, et al Protease inhibitor monotherapy in controlling HIV-1 shedding in the male genital tract. Clin Microbiol Infect 2015 10 7 pii: S1198-743X(15)00898-8. 10.1016/j.cmi.2015.09.028. [Epub ahead of print] 26454060

[34] Gutmann C, Cusini A, Guntard H, Fux A, Hirschel B, Descorted L, et al Randomized controlled study demonstrating failure of LPV/r monotherapy in HIV: the role of compartment and CD4-nadir. AIDS 2010;24:2347–2354. 10.1097/QAD.0b013e32833db9a1 20802298

[35] Arenas-Pinto A, Stöhr W, Jäger HR, Haddow L, Clarke A, Johnson M, Chen F, Winston A, Godi C, Thust S, Trombin R, Cairns J, Solanky BS, Golay X, Paton NI; PIVOT Neurocognitive sub-study Team. Neurocognitive function and neuroimaging markers in virologically suppressed HIV-positive patients randomized to ritonavir-boosted protease inhibitor monotherapy or standard combination ART: A cross-sectional substudy from the PIVOT Trial. Clin Infect Dis. 2016 5 3. pii: ciw279. [Epub ahead of print] 10.1093/cid/ciw279 PMC4928386 27143662

[36] Clarke A, Johanssen V, Gerstoft J, Clotet B, Ripamonti D, Murungi A, et al Analysis of neurocognitive function and CNS endpoints in the PROTEA trial: darunavir/ritonavir with or without nucleoside analogues. J Int AIDS Soc. 2014;17(4 Suppl 3):19526 10.7448/IAS.17.4.19526 25394035 PMC4224914

[37] Vinuesa D, Parra-Ruiz J, Chueca N, Alvarez M, Muñoz-Medina L, Garcia F, et al Protease inhibitor monotherapy is not associated with increased viral replication in lymph nodes. AIDS 2014;28(12):1835–1837. 10.1097/QAD.0000000000000312 24835357

[38] Van Leeeuwen E, Prins JM, Jurriaans S, Boer K, Reiss P, Repping S, et al Reproduction and fertility in human immunodeficiency virus type-1 infection. Human Reproduction Update 2007;13:197–206. 17099206 10.1093/humupd/dml052

[39] Lambert-Niclot S, Poirot C, Tubianan R, Houssaini A, Soulié C, Dominguez S, et al Effect of antiretroviral drug on the quality of semen. J Virol. 2011;83:1391–1394. 10.1002/jmv.22119 21678443

[40] Moyle G. Mechanisms of HIV and nucleoside reverse transcriptase inhibitor injury to mitochondria. Antivir Ther. 2005;10 Suppl 2:M47–52 16152705

[41] Moyle GJ, Data D, Mandalia S, Morlese J, AsboeD, Gazzard BG. Hyperlactataemia and lactic acidosis during antiretroviral therapy: relevance, reproducibility and possible risk factor. AIDS 2002;16:1341–1349. 12131210 10.1097/00002030-200207050-00005

[42] Molina I, Gonzalvo MC, Clavero A, López-Ruz MA, Mozas J, Pasquau J, et al Assisted reproductive technology and obstetric outcome in couples when the male partner has a chronic viral disease. Int J Fertil Steril. 2014;7:291–300. 24520499 PMC3901182

